# Comparison of Fat Suppression Sequences T2 Weighted Two-Point Dixon and Short-Tau Inversion Recovery in Magnetic Resonance Imaging of Lumbar Spine: An Observational Study

**DOI:** 10.31729/jnma.8899

**Published:** 2025-03-31

**Authors:** Umesh Khanal, Ramswarth Sah, Sushil Kumar Mahato, Shailendra Katwal, Ghanshyam Gurung

**Affiliations:** 1 Department of Radiology and Imaging, Tribhuvan University Teaching Hospital, Maharajgunj Medical Campus, Maharajgunj, Kathmandu, Nepal; 2 Department of Radiology and Imaging, National Trauma Center, Kathmandu, Nepal

**Keywords:** *contrast*, *fat*, *intensity*, *noise*, *signal*

## Abstract

**Introduction:**

Magnetic Resonance Imaging is a common diagnostic tool used to evaluate various clinical conditions. Different fat suppression techniques such as Short Tau Inversion Recovery and Dixon are employed to enhance diagnostic accuracy. The choice of fat suppression sequence varies based on availability and performance. This study aimed to compare Contrast Ratio and Contrast-to-Noise Ratio of Short Tau Inversion Recovery and Dixon technique.

**Methods:**

This observational cross-section study was performed in the Department of Radiology from 8 September 2023 to 7 September 2024 after the approval by the Institutional Review Committee (Reference number: 151/080/081(6-11)E2). Using a complete census method during the study period, MRI-lumbar spines of 384 adult patients were included in the study. Signal intensity values of the lesion, adjacent normal tissue, and noise were recorded for calculation of Contrast Ratio and Contrast-to-Noise Ratio.

**Results:**

Among 384 cases, 191 (49.74%) were male and 193 (50.26%) were female with median age 46 (IQR: 35-60) years. The median value of contrast ratio and contrast-to-noise ratio were 0.31 (IQR: 0.14-0.50) and 8.74 (IQR: 3.82-15.50) respectively for STIR sequence while median value of contrast ratio and contrast-to-noise ratio were 0.44 (IQR: 0.19-0.74) and 11.95 (IQR: 5.38-23.18) respectively for Dixon sequence, higher than Short Tau Inversion Recovery.

**Conclusions:**

Contrast Ratio and Contrast to Noise Ratio were found higher and background noise lower for Dixon compared to Short Tau Inversion Recovery.

## INTRODUCTION

Fat suppression is a Magnetic Resonance Imaging (MRI) technique in which signal from adipose tissue is suppressed to better visualize contrast uptake by body's tissues, reduce chemical-shift artifact, characterize certain lesions and improve to increase the diagnostic confidence in lumbar spine diseases.^1,^ ^2^ Due to short relaxation times, fat has high signal that helps to characterize lesions but also causes artifacts such as ghosting, chemical shift and mask subtle contrast difference in non-fatty tissue by filling the dynamic range of receiver mostly with fat signal and hide contrast enhancing tumor by surrounding it.^3^

Fat suppression can be achieved in different ways. But, due to empirical and practical knowledge gap, incomplete fat suppression and anatomical deterioration are common in fat suppressed images.^4-7^ Thus, the aim of our study was to compare the effectiveness of Fat Suppression Sequences: T2-weighted Two-point Dixon and Short Tau Inversion Recovery (STIR) in MRI of lumbar Spine images in terms of Contrast Ration (CR) and Contrast to Noise Ratio (CNR).

## METHODS

This observational cross-section study was conducted among 384 patients of age group 18-84 years visiting the Department of Radiology and Imaging, Tribhuvan University Teaching Hospital, Nepal for MRI of lumbar spine. A total population sampling was done which included images of all patients between September 08, 2023 and September 07. The study included all patients with lower back pain or radiculopathy necessitating lumbar spine MRI examinations and referred for lumbar spine MRI examinations in the Department of Radiology and Imaging excluding infants, children, and individuals with post-instrumentation implants.

Before commencing the study, ethical approval was obtained from the Institutional Review Committee of Institute of Medicine, Tribhuvan University (Reference number: 151/080/081(6-11)E2). Informed consent was obtained from the patients or the legal guardian before enrollment in the study. To minimize sedation time, the extra fat suppression technique (Dixon) was not taken in case of pediatric patients.

Data were acquired using the 1.5T Magnetom Amira Siemens MRI scanner. All patients underwent thorough screening by departmental guidelines to identify presence of any ferromagnetic materials and patients were positioned in the 1.5T Magnetom Amira Siemens MRI scanner. The routine department imaging sequences including Short Tau Inversion Recovery (STIR) was followed by an additional T2 Dixon sequence in sagittal plane using predefined parameters (Table 1).

After completion of scanning for all sequences, sagittal images of T1 weighted, T2 weighted, STIR and T2 Dixon so obtained were evaluated. Any abnormality and signal changes in lumbar spine were first observed and measurement was taken. The symmetrical (same size) of region of interest (ROI) was drawn in normal region, on the lesion (Modic changes) noted on body of lumbar spine and also on the background to measure signal intensity (SI) which was represented by mean/SD on lumbar spine images of STIR and T2 Dixon sequences respectively. Finally, contrast ratio (CR) and contrast to noise ratio (CNR) were calculated using the following formulae: CR = (S_Ilesion_ - S_Inormal_)/S_Inormal_ and CNR = (SI_lesion_ - S_Inormal_)/S_Inoise_ Where, CR is contrast ratio, CNR is contrast-to-noise ratio, SIlesion is signal intensity from lesion, SInormal is signal intensity from normal tissue and SInoise is signal intensity from background noise.^8^

A predesigned pro-forma was used for recording socio-demographic variables, SI, CR and CNR. Data were entered in Microsoft Excel and then exported to and analysed using Statistical Package for the Social Sciences (SPSS), version 27 (IBM Corp., Armonk, N.Y., USA) for descriptive analysis.

**Table 1:** Magnetic resonance imaging parameters for short-tau inversion recovery and Dixon sequences.

Parameters	DIXON	STIR
Repetition Time (ms)	3970	5530
Echo Time (ms)	75	70
Echo Train Length	15	20
Number of acquisitions	1	1
Concatenations	1	1
No. of slices	15	15
Slice thickness(mm)	4	4
Phase encoding direction	Head to feet	Head to feet
Acquisition time	2min 20 sec	2 min 30 sec
Phase oversampling (%)	70	70
Feild of View	300 X 300	300 X 300
Inversion time (ms)	NA	160

## RESULTS

A total of 384 patients were included in the study. Age of the patient ranged from 18 years to 84 years with median age 46 (IQR: 35-60) years. Among the total 384 patients, 191 (49.74%) were males and 193 (50.26%) were female (Table 2).

The median SI value in normal tissue and in lesion were 94.80 (IQR: 76.55-117.5) and 85.30 (IQR: 62.07-120.77) respectively in the image of STIR sequence while they were found 73.65 (IQR: 56.70-100.6) in normal tissue and 65.90 (IQR: 43.60-103.07) in the lesion in the image of Dixon sequence. The median noise value in the image of STIR sequence was 3.20 (IQR: 2.30-4.70) while it was 2.50 (IQR: 1.70-3.80) in case of image of Dixon sequence (Table 3).

**Table 2:** Distribution of study population according to sex and age group (n = 384).

Sex	Frequency (%)	18-28 years	28-38 years	38-48 years	48-58 years	58-68 years	68-78 years	≥78 years
Male	191 (49.74)	26 (13.60)	39 (20.40)	31 (16.20)	35 (18.30)	48 (25.10)	10 (5.20)	2 (1.00)
Female	193 (50.26)	17 (8.80)	33 (17.10)	57 (29.50)	42 (21.80)	26 (13.50)	12 (6.20)	6 (3.10)

**Table 3:** Distribution of signal intensity in the images of STIR and Dixon sequence, expressed in median (IQR).

STIR sequence			Dixon sequence		
SI_normal_	SI_lesion_	SI_noise_	SI_normal_	SI_lesion_	SI_noise_
94.80 (76.55-117.50)	85.30 (62.07-120.77)	3.20 (2.30-4.70)	73.65 (56.70-100.60)	65.90 (43.60-103.07)	2.50 (1.70-3.80)
SI_normal_ = Signal Intensity on normal Tissue, SI_lesion_ = Signal Intensity on Lesion, SI_noise_ = Signal Intensity on Image background					

The Median values of CR for images obtained by STIR and Dixon sequences were 0.31 (IQR: 0.14-0.50) and 0.44 (IQR: 0.19-0.74) respectively. Similarly, the median value of CNR for the images obtained by STIR and Dixon sequences were found to be 8.74 (IQR: 3.82-8.73) and 11.95 (IQR: 5.38-23.18) respectively. The median value for background noise was 2.50 (IQR: 1.70-3.80) for images obtained by Dixon sequence while that for images obtained by STIR sequence, 3.20 (IQR: 2.30-4.70) A significant differences in CR, CNR and background noise of the images obtained by STIR and Dixon sequences (p-value <0.001 for CR, CNR and background noise and z-value 9.68 for CR, 10.14 for CNR and 14.29 for background noise) (Table 4).

**Table 4:** Distribution of CR, CNR and noise in the images of STIR and Dixon sequence, expressed in median (IQR).

CR_Dixon_	CR_stir_	CNR_Dixon_	CNR_stir_	Background Noise_Dixon_	Background Noise_STiR_
0.44 (0.19-0.74)	0.31 (0.14-0.50)	11.95 (5.38-23.18)	8.74 (3.82-8.73)	2.50 (1.70-3.80)	3.20 (2.30-4.70)

CR = Contrast Ratio, CNR = Contrast-to-Noise Ratio, CR_Dixon_ = Contrast Ratio of the images obtained by DIXON sequence, CR_STIR_ = Contrast Ratio of the images obtained by STIR sequence, CNR_Dixon_ = Contrast-to-Noise Ratio of the images obtained by DIXON sequence, CNR_STIR_ = Contrast-to-Noise of the images obtained by STIR sequence

## DISCUSSION

Magnetic Resonance Imaging provides standardized, non-invasive assessments of spinal pathologies and thus is one of the most important imaging modality regarding the diagnosis and management of the spinal pathologies.^1, 9^ Fat suppression technique is crucial to better visualize uptake of contrast material by body's tissues, reduce chemical-shift artifact, characterize certain lesions (modic changes, degenerative disc disease, marrow edema) and improve to increase the diagnostic confidence in lumbar spine diseases.^1,^ ^2^ Understanding the different fat-suppression options allows radiologists to adopt the most appropriate technique for their clinical practice.^10^

Our study aimed to compare the degree of fat suppression in terms of CR, CNR and background noise in the images produced by Dixon sequence and that produced by STIR sequence. For quantitative evaluation of fat suppression and lesion conspicuity, the calculated median CR-value for images obtained by STIR and Dixon sequences were found 0.31 (IQR: 0.14-0.50) and 0.44 (IQR: 0.19-0.74) respectively and the median CNR calculated for the images obtained by STIR and Dixon sequences were found 8.74 (IQR: 3.82-8.73) and 11.95 (IQR: 5.38-23.18) respectively. There was a difference between the CR values of images obtained by STIR sequence and Dixon sequence along with the CNR values of images obtained by STIR sequence and Dixon sequence.

In our study, CR and CNR were found to be higher and background noise lower for Dixon compared to Short Tau Inversion Recovery.

Wohlgemuth et al. conducted a comparative study measuring CR and CNR in 27 patients and found that multipoint Dixon technique shows superior SNR and CNR than STIR in the delineation of traumatic fractures.^11^ Similarly, Lee S et al. conducted a similar comparative study in 79 patients and found T2 Dixon as superior to Spectral Attenuated Inversion Recovery (SPAIR) in quantitative parameters (better CR and CNR, p-value <0.01) which also support our study results.^1^ In spite of small sample size in the above two studies, they found similar result to our study findings which highlight the superiority of Dixon sequence as fat suppression technique.

Likewise, Zadig P et al. in 2023 conducted a study entitled Pediatric whole-body magnetic resonance imaging: comparison of STIR and T2 Dixon sequences in the detection and grading of high signal bone marrow changes and found that 90% of high signal bone marrow areas identified on a 1.5-T whole-body MRI were seen on both STIR and water-only T2W Dixon, while 5% were seen on STIR only and 5% were seen on T2W Dixon only. This result may be due to difference in inclusion criteria.^3^

Low RN et al. conducted a comparative study to evaluate a prototype fast spin-echo (FSE) tripleecho Dixon (FTED) technique for T2-weighted spine imaging with and without fat suppression compared to conventional T2-weighted fast recovery (FR) FSE and short-tau inversion recovery (STIR) imaging and found the FTED water images demonstrated less motion, better anatomic sharpness and less CSF, however, there was no difference in fat suppression and chemical shift artifact.^12^

Ma J et al. conducted a comparative study and found mean scores 3.2 and 2.1 for the uniformity of fat suppression, and 3.0 and 2.0 for the lesion conspicuity for the fast Three Point Dixon and the chemical shift selective (CHESS) fat suppression techniques, respectively. In spite of limited sample size 27, this study's result also showed fast three Point Dixon technique statistically superior to the CHESS technique (P < 0.0005).^13^

In our study, the median background noise measured was found 2.50 (IQR: 1.70-3.80) for Dixon sequence and that for STIR sequence was found 3.20 (IQR: 2.304.70) This quantitative parameter also highlights Dixon has better fat suppression technique in comparison to STIR technique. This statement was also supported by a similar study performed by Dalto VF et al.^14^

This study excluded all the patients under 18 years of age as well as those patients who required sedation during the examination. Thus, it is not possible to generalize this study results in patients below 18 years of age. Additionally, this study did not compare SI, CR and CNR in different clinical condition like disc degenerative disease, modic changes and marrow edema separately for evaluation of effectiveness of STIR and Dixon as fat suppression technique.

## CONCLUSIONS

The quantative parameter suggests a better fat suppression by Dixon sequence which is comparable to other studies which shows higher degree of fat suppression along with less total scan time by Dixon sequence in comparison to that of STIR sequence for the evaluation of lumbar spine lesions.
